# The Significance of an Increased Beta-Hydroxybutyrate at Presentation to the Emergency Department in Patients with Diabetes in the Absence of a Hyperglycemic Emergency

**DOI:** 10.1155/2019/7387128

**Published:** 2019-10-10

**Authors:** Barbara Depczynski, Alexandra Tze Kiu Lee, Wayne Varndell, Angela L. Chiew

**Affiliations:** ^1^Endocrinology Department, Prince of Wales Hospital, Randwick, NSW, Australia; ^2^Emergency Department, Prince of Wales Hospital, Randwick, NSW, Australia

## Abstract

The significance of hyperketonemia in adults with diabetes presenting to the emergency department with acute illness, not due to a diabetic hyperglycemic emergency, has not been well characterized. Adult patients with diabetes presenting to the emergency department who had venous blood gas and beta-hydroxybutyrate levels measured whilst in the emergency department were retrospectively evaluated for the relationship between BHB and clinical outcomes. Over 6 months, 404 patients with diabetes had at least one beta-hydroxybutyrate level measured in the emergency department. There were 23 admissions for diabetic ketoacidosis (DKA) or hyperosmolar state. Of the remainder, 58 patients had a beta-hydroxybutyrate ≥ 1 mmol/L; this group had a higher glucose at presentation (19.0 (8.8) versus 10.4 (9.9) mmol/L), higher HbA1c (8.8 (5.4) versus 8.0 (3.3)%), lower bicarbonate (22.6 (6.2) versus 24.8 (4.7) mmol/L), and higher anion gap (14.8 (6.1) versus 12.6 (4.2)) than had those with BHB < 1 mmol/L. There was no association between the presence of ketosis and the length of stay (4.2 (7.3) versus (3.0) (7.2) days). Acute illness in those with diabetes associated with ketosis in the absence of DKA is associated with worse glycaemic control than in those without ketosis. Ketosis may represent an intermediate state of metabolic dysregulation rather than being associated with a more severe acute illness, as suggested by no relationship between BHB and length of stay.

## 1. Introduction

Ketone bodies—beta-hydroxybutyrate (BHB), acetoacetate, and acetone—are an important energy source during energy restriction. They are predominately produced from fatty acids by the liver [1]. The prevalence and clinical significance of ketosis in acute illness in adult patients with diabetes, in the absence of a hyperglycemic emergency, have not been well defined. The degree of BHB elevation with hyperglycemic emergencies has been well characterised, with BHB level > 3 mmol/L in children or >3.8 mmol/L in adults being presumptive evidence of diabetic ketoacidosis [2]. Normally, circulating BHB levels are below 0.5 mmol/L [1], but with starvation, levels can rise up to 6 mmol/L [3]. A study performed in a paediatric population presenting to the ED found an association between ketone levels and fever, and ketone levels were higher in children who were admitted [4]. This suggests that ketosis occurs as part of a stress response to illness. BHB, in addition to its traditional role as a fuel source during fasting, also has an important role as a signalling link between the metabolic and immune systems [1]. There is a paucity of information about the expected degree of ketosis in adults with acute illness. Defining the expected degree of ketosis with acute illness has become more important given the ability to undertake point-of-care BHB testing [5] and that sodium glucose cotransporter type 2 inhibitors (SGLT2i) may precipitate euglycaemic DKA with intercurrent illness [6].

The objectives of this study are to examine whether ketosis is associated with greater metabolic derangement by examining the relationship between BHB and glucose, HCO_3_, anion gap (AG), and base excess (BE) and whether ketosis is associated with a more severe acute illness, as determined by an increased length of stay, in-hospital mortality, or higher lactate at presentation. In order to explore whether BHB may be part of a systemic stress response, the frequency of systemic inflammatory response syndrome (SIRS) [7] according to the presence of ketosis will be assessed.

## 2. Methods

### 2.1. Design and Setting

The study population consisted of adult patients attending the ED of Prince of Wales Hospital, a tertiary referral teaching hospital in an urban area of Australia. Patients were eligible if a point-of-care (POC) BHB measurement and venous blood gas (VBG) measurement had been performed at presentation to the ED. BHB testing had been encouraged in the ED due to reports of euglycaemic DKA, given the increased use of sodium glucose cotransporter type 2 inhibitors (SGLT2i) in the community [6].

### 2.2. Data Extraction

Demographic data, as well as clinical and laboratory variables obtained at the time of the presentation and captured in the electronic medical record (Cerner Millennium), were retrospectively retrieved and used for this analysis. We collected epidemiological information (age, gender, length of stay, and admitting team), medical history (diabetes management at presentation and presence of comorbidities to determine the Charlson score [8], as well as the presence of other contributors to risk for ketosis including reduced oral intake or ethanol intake), biochemical characteristics (glucose, BHB, lactate, anion gap (AG), base excess (BE), estimated glomerular filtration rate (eGFR), and white cell count (WCC)), and clinical observations at presentation (heart rate, blood pressure, and temperature). VBG measurements were performed using ABL800 Flex (Radiometer Medical ApS, Denmark). BHB levels were measured using a POC machine (Nova Biomedical Corporation, USA), which has been well validated against laboratory measurement [5]. AG was calculated by subtracting the sum of chloride and bicarbonate concentration from the sum of sodium and potassium concentration [9]. The presence or absence of diabetes was as documented in the admission or discharge summary and included new diagnoses made during that admission. Reduced oral intake, vomiting, or acute ethanol intake was recorded if documented in the medical record. DKA and HHS were defined by Kitabchi et al. [10] with the triad of metabolic acidosis, hyperglycemia, and ketosis, although the measurement of BHB was performed using POC BHB level rather than by laboratory measurement [2, 3]. Euglycaemic DKA was defined by Kitabchi et al. [10] but again with BHB measured using a POC machine.

Systemic inflammatory response syndrome (SIRS) is an inflammatory process, independent of its cause seen in a range of conditions including sepsis [7]. It was defined as two or more of the following: temperature < 36°C or >38°C, respiratory rate > 20 breaths/min, heart rate > 90 beats/minute, WCC < 4 × 10^9^/L or >12 × 10^9^/L, or >10% band forms [7]. Data to calculate Quick Sequential (sepsis-related) Organ Failure Assessment (qSOFA) were also collected, with 2 or more of the following variables considered as a positive score: respiratory rate ≥ 22/minute, systolic blood pressure ≤ 100 mmHg, or altered mentation [11]. The presence of preexisting comorbid conditions was defined by the Charlson comorbidity index score [8].

### 2.3. Analysis

Since BHB levels not higher than 0.9 mmol/L were seen in patients on SGLT2i [12], BHB does not exceed 1 mmol/L, irrespective of glucose in the absence of intercurrent illness in T1DM [13], and hyperketonemia has previously been defined at a threshold of 1 mmol/L regardless of diabetes status [3], patients were grouped according to whether BHB level was <1 mmol/L (low-BHB group or no-ketosis group), or BHB was ≥1 mmol/L without DKA (high-BHB group or ketosis group), or the presence of DKA or HHS.

Data were expressed as the medians and interquartile ranges (IQRs), as the mean and standard deviation, or as percent. Statistics were obtained using SPSS version 25 (IBM SPSS Statistics for Windows, version 25.0 Armonk, NY, IBM Corporation). The Kruskal-Wallis *H* test was used for comparisons between groups for continuous variables. Where there were significant differences, post hoc analysis was performed using Dunn's procedure with a Bonferroni adjustment. For categorical variables, a *χ*^2^ test was performed. Ethics approval was obtained from South Eastern Sydney Local Health District Human Research Ethics Committee, who waived the need for consent.

## 3. Results

### 3.1. Number and Characteristics of Participants

Over a 6-month period in 2018, 404 patients with diabetes presenting to the ED had at least one VBG level and one BHB level measured whilst in the ED. There were 23 diabetes emergencies in total, including DKA in 12 patients with T1DM, DKA due to a SGLT2i in a patient with T2DM, DKA in 2 patients with T2DM, and DKA in 2 patients with diabetes due to exocrine pancreatic disease; HHS was present in 6 cases. Three hundred twenty-three patients had BHB below 1 mmol/L, and 58 patients had BHB ≥ 1 mmol/L but without DKA.

The median age was 71.7 (range 16.3-102.3) years. 57.3% of the cohort were men. The median length of stay (LOS) was 3.0 (range 0.5-77.8) days. 76% of the cohort were admitted to the hospital, the majority under a medical team, with both the low- and high-ketone groups containing a similar proportion of surgical patients (12.4 and 8.6%). For the whole cohort, median glucose was 12.6 (IQR, 8.5-19.4) mmol/L, median lactate was 1.9 (IQR 1.5-2.8) mmol/L, and median BHB was 0.2 (IQR 0.1-0.6) mmol/L.

Demographic features of the cohort, stratified for ketone and diabetic emergency status, are shown in Table 1. Those presenting with DKA were younger (median age was 48.9 years, but there were no differences between the other groups). T1DM was the most frequent type of diabetes in the DKA group, but otherwise, the types of diabetes did not vary between the low- and high-ketone groups. The frequency of insulin use did not differ across the groups, and the overall use of SGLT2i was low, 3.4 and 5.2% of the low- and high-ketone groups, respectively. The burden of preexisting comorbidities, as reflected by the Charlson score, was similar across the groups.

### 3.2. Relationship between BHB and Metabolic State

There was a positive correlation between BHB and glucose (*r* = 0.349, *p* = 0.0005). With the exclusion of those with DKA or HHS, there was a negative correlation between BHB and HCO_3_ (*r* = ‐0.317, *p* = 0.0001) and a positive correlation between BHB and anion gap (*r* = 0.253, *p* = 0.0001). HCO_3_ and BE were the lowest in the DKA group, but they were lower in the ketosis group as compared with the no-ketosis group. Similarly, AG was the highest in the DKA group, but it was higher in the ketosis group versus no-ketosis group. HbA1c was the highest in those presenting with HHS (14.0 (9.1) %); the HbA1c of the ketosis group was higher than that of the no-ketosis group (8.8 (5.4) versus 8.0 (3.3) %, *p* < 0.05). The glucose at presentation in those with ketosis was higher than that in those without ketosis (19.0 (8.8) versus 10.4 (9.9) mmol/L, *p* < 0.05). The median BHB of those on insulin at admission was no different to that of those not on insulin at admission (0.2 (0.4) versus 0.2 (0.3) mmol/L), and similarly, BHB was not different for those on or not on a SGLT2i (0.2 (0.3) versus 0.3 (0.8) mmol/L).

### 3.3. Severity of Acute Illness and Other Relationships with BHB

The LOS did not differ between the ketone groups, as shown in Table 1 (median LOS for the low-ketone group was 3.0 (7.2) days, for the high-ketone group 4.2 (7.3) days, and for the DKA group 3.2 (4.1) days). There were no differences in inpatient mortality across the ketone groups. LOS correlated with lactate (*r* = 0.125, *p* = 0.013) but not with BHB (*r* = 0.063). The frequency of SIRS was the lowest in those free of ketosis (22.6%), with a similar frequency in the other groups (41.4-50%). The median BHB of the high-ketone group was 0.4 (0.9) mmol/L versus 0.2 (0.3) mmol/L in the no-ketosis group (*p* < 0.01). The qSOFA score did not differ between the groups. WCC was the highest in those with ketosis or DKA, as shown in Table 1. The frequency of documented acute ethanol intake was the highest amongst those presenting with DKA (16.7%) and the next highest in those with ketosis (8.6%). In none of these cases was ketosis ascribed to a diagnosis of alcoholic ketoacidosis. Patients with ketosis or DKA were likely to have reduced oral intake or vomiting as documented at presentation, as shown in Table 1.

## 4. Discussion

Hyperketonemia defined as BHB ≥ 1 mmol/L, in the absence of a diagnosis of DKA or HHS, was present in 14% of this series of patients with diabetes attending an adult emergency department who had VBG and BHB levels measured. Results of HCO_3_, AG, and BE for those with ketosis were intermediate between those without ketosis and those with DKA. Those with ketosis had poorer prior glycaemic control, as reflected by a higher HbA1c than in those without ketosis, and had greater acute hyperglycemia on presentation. These results suggest that ketosis in the absence of a diabetic emergency reflects in a vulnerable patient greater metabolic decompensation with acute illness rather than severity of acute illness as reflected by LOS, which was not different between the low- and high-ketone groups.

The rate of ketosis in our cohort is lower than that seen in acutely unwell children without diabetes presenting to the ED, where those with BHB ≥ 1.2 mmol/L or higher comprised 34.4% of the cohort [4]. However, the frequency of hyperketonemia in ED in our series was higher than that seen amongst patients without diabetes fasting for surgical procedures, where it was only 3% [14]. This difference in the rate of ketosis may suggest that fasting or reduced oral intake alone is not responsible for the development of ketosis. Adults with diabetes, especially if poorly controlled, or with hyperglycemia may warrant undergoing BHB testing in the presence of acute illness. Amongst those with type 1 diabetes and diabetes due to pancreatic disease, where there is hyperglycemia or concern for diabetic ketoacidosis due to metabolic acidosis or symptoms of ketosis such as nausea and vomiting, BHB testing is warranted. Given the severity of outcomes with SGLT2i-induced DKA in prior reports [6], BHB testing in any patient presenting acute illness on this class of drug would be prudent even though the ketone level in those on this drug did not differ from those not on a SGLT2i. In the presence of an unexplained metabolic acidosis, BHB testing may help with the diagnostic workup.

Those with ketosis, DKA, or HHS had poorer prior glycaemic control, as reflected by higher HbA1c, and had greater hyperglycemia on presentation. Hyperglycemia and T2DM are associated with higher BHB levels in the absence of acute illness [15]. We did not see a relationship between BHB and LOS, suggesting that the occurrence of ketosis does not reflect a more severe acute presentation. In support of this, lactate correlated with LOS, but there was no relationship between BHB and LOS. We had assessed lactate as a surrogate marker or a predictor of poorer hospital outcomes and more severe illness at presentation [16], but we did not collect data on whether type A or B hyperlactatemia may have been present.

Aside from its role as an alternate fuel source, there is also evidence that BHB provides a signalling link between the metabolic and immune systems [1]. There is substantial evidence for an increase in inflammation occurring under conditions of hyperketonemia [17]. Increased levels of the ketone body acetoacetate result in increased oxidative stress in red blood cells as shown by increased lipid peroxidation and reduced glutathione [18]. Hyperketonemia can also increase tumour necrosis factor alpha secretion [19] and increase monocyte adhesion to vascular endothelium [20]. Acetoacetate has been shown to upregulate NADPH oxidase 4, contributing to oxidative stress of endothelial cells [21]. Although we have shown an association between BHB and SIRS, our method limits any further interpretation. The SIRS response did not reflect sepsis given that the qSOFA scores were not different between the groups. Potentially, the components of SIRS in our series may have been abnormal due to volume contraction due to reduced oral intake or an increased respiratory drive may have arisen in response to an increased anion gap. However, in a paediatric cohort fasting for surgery, lower ketosis was associated with a better mean arterial pressure [22], suggesting that ketonemia in itself is potentially harmful to the haemodynamic state. An inverted U-shaped relationship between BHB and physiological effects has been proposed with mild elevation being beneficial and higher levels being harmful [23].

The frequency of ketosis varied with the acute nutritional state. This finding is similar to that from a paediatric ED where a strong correlation between BHB levels and reduced oral intake was found [4]. It is unclear whether the development of ketosis contributed to reduced intake and vomiting in our ketosis cohort. Although no patient in our series was given a diagnosis of alcoholic ketoacidosis, a high rate of documented acute ethanol intake prior to presentation in the DKA and ketosis groups was seen. It has been suggested that cases of alcoholic ketoacidosis may be missed or underreported in the ED with presentation ascribed to other diagnoses such as acute gastritis [24], and so BHB testing may be useful if this is suspected.

Our study has several limitations, particularly that the data are observational, limiting any conclusions on causality. Patients with diabetes that remained undiagnosed may not have been included. It is unknown whether the BHB result influenced glycaemic management and, thus, outcomes. LOS as a reflection of severity of presentation is open to confounders such as comorbidities; however, we attempted to address this by assessing the Charlson score, which did not differ between the groups. SIRS as a marker of inflammation or stress response is nonspecific [7], and as discussed, the score may instead reflect physiological responses to the metabolic derangements. A further limitation is that acetoacetate measurement was not assessed. Acetoacetate measurement can be helpful in the assessment of patients with increased BHB and acute illness [17].

In conclusion, this study shows that ketosis may be seen with acute illness in adults with diabetes. Ketosis may represent an intermediate stage of metabolic derangement in association with acute illness. Ketosis is associated with the presence of SIRS at hospital presentation, and whether ketosis is part of an acute stress response requires further exploration.

## Figures and Tables

**Table 1:** Characteristics of patients with diabetes presenting to the emergency department, stratified by beta-hydroxybutyrate category or presence of hyperglycemic emergency.

	Beta-hydroxybutyrate < 1 mmol/L (low-ketone group)	Beta-hydroxybutyrate ≥ 1 mmol/L (ketosis without DKA)	DKA	HHS
Number (%)	323 (79.9)	58 (14.4)	17 (4.2)	6 (1.5)
Age year median (IQR)^∗^	72.2 (22.5)	63.4 (34.3)	48.9 (44.6)^#^	77.1 (39.6)
Men (%)^∗^	57.6	60.3	61.1	0
Type of diabetes (%)^∗^				
T1	12.1	19.0	72.2	0
T2	83.9	72.4	16.7	100
T3	2.8	5.2	11.1	0
Use of SGLT2i at presentation (%)	3.4	5.2	0	0
Use of insulin at presentation (%)	52.8	53.4	81.3	33.3
Admitting team				
Surgery	10.5	10.3	-	-
Cardiac	12.4	8.6	-	-
Respiratory	7.4	5.2	-	-
Geriatric	12.1	17.2	-	33.3
Other medical	30.0	31.0	100	66.7
Discharge from ED	25.7	25.9	-	-
Length of stay median (IQR)	3.0 (7.2)	4.2 (7.3)	3.2 (4.1)	3.5 (10.5)
In-hospital death percent	5.1	5.3	4.9	8.6
Reduced oral intake at presentation^∗^ (%)	15.2	32.8	55.6	16.7
Vomiting at presentation^∗^	13.6	19	72.2	0
Acute ethanol at presentation^∗^ (%)	3.4	8.6	16.7	0
SIRS^∗^ (%)	22.6	41.4	44.4	50.0
qSOFA (%) *p* = 0.412	1.9	5.2	0	0
Charlson score Arbitrary units (IQR)	3 (2)	2.8 (2.0)	2.1 (1.5)	1.4 (1.5)
Glucose^∗^ (mmol/L) Median (IQR)	10.4 (9.9)	19 (8.8)^#^	29.8 (14.4)^#^	30.4 (12)^#^
Beta-hydroxybutyrate (mmol/L) Median (IQR)	0.2 (0.3)	2.1 (1.5)	6.3 (0.4)	1.5 (3.0)
Lactate (mmol/L) Median (IQR)	2.3 (1.8)	2.4 (2.2)	2.1 (0.2)	1.9 (1.0)
eGFR (mL/min/1.73 m^2^) Median (IQR)	57.5 (53)	65 (58)	75 (36)	55 (48)
Bicarbonate^∗^ (mmol/L) Median (IQR)	24.8 (4.7)	22.6 (6.2)^#^	12.3 (5.0)^#^	24.8 (9.4)
Anion gap^∗^ Median (IQR)	12.6 (4.2)	14.8 (6.1)^#^	27.1 (6.0)^#^	17.2 (5.9)
Standard base excess^∗^	0.6 (4.3)	-1.8 (6.5)^#^	-14 (5.6)^#^	0.3 (8.9)
HbA1c^∗^ (%) Median (IQR)	8.0 (3.3)	8.8 (5.4)^#^	9.4 (1.3)^#^	14.0 (9.1)^#^
WCC^∗^ (cells/*μ*L) Mean (IQR)	10.2 (5.9)	12.7 (6.1)^#^	15.5 (9.0)^#^	10.5 (4.6)

^∗^ *p* < 0.05 across groups. ^#^*p* < 0.05 compared with the no-ketosis group.

## Data Availability

The data used to support the findings of this study are included within the article.

## References

[1] Puchalska P., Crawford P. A. (2017). Multi-dimensional roles of ketone bodies in fuel metabolism, signaling, and therapeutics. *Cell Metabolism*.

[2] Sheikh-Ali M., Karon B. S., Basu A. (2008). Can serum *β*-hydroxybutyrate be used to diagnose diabetic ketoacidosis?. *Diabetes Care*.

[3] Laffel L. (1999). Ketone bodies: a review of physiology, pathophysiology and application of monitoring to diabetes. *Diabetes/Metabolism Research and Reviews*.

[4] O'Donohoe P. B., Kessler R., Beattie T. F. (2006). Exploring the clinical utility of blood ketone levels in the emergency department assessment of paediatric patients. *Emergency Medicine Journal*.

[5] Ceriotti F., Kaczmarek E., Guerra E. (2015). Comparative performance assessment of point-of-care testing devices for measuring glucose and ketones at the patient bedside. *Journal of Diabetes Science and Technology*.

[6] Meyer E. J., Gabb G., Jesudason D. (2018). SGLT2 inhibitor–associated euglycemic diabetic ketoacidosis: a South Australian clinical case series and Australian spontaneous adverse event notifications. *Diabetes Care*.

[7] Bone R. C., Balk R. A., Cerra F. B. (1992). Definitions for sepsis and organ failure and guidelines for the use of innovative therapies in sepsis. *Chest*.

[8] Charlson M. E., Pompei P., Ales K. L., MacKenzie C. R. (1987). A new method of classifying prognostic comorbidity in longitudinal studies: development and validation. *Journal of Chronic Diseases*.

[9] Kraut J. A., Madias N. E. (2007). Serum anion gap: its uses and limitations in clinical medicine. *Clinical Journal of the American Society of Nephrology*.

[10] Kitabchi A. E., Umpierrez G. E., Miles J. M., Fisher J. N. (2009). Hyperglycemic crises in adult patients with diabetes. *Diabetes Care*.

[11] Seymour C. W., Liu V. X., Iwashyna T. J. (2016). Assessment of clinical criteria for sepsis: for the Third International Consensus Definitions for Sepsis and Septic Shock (Sepsis-3). *JAMA*.

[12] Ferrannini E., Baldi S., Frascerra S. (2016). Shift to fatty substrate utilization in response to sodium–glucose cotransporter 2 inhibition in subjects without diabetes and patients with type 2 diabetes. *Diabetes*.

[13] Wallace T. M., Meston N. M., Gardner S. G., Matthews D. R. (2001). The hospital and home use of a 30-second hand-held blood ketone meter: guidelines for clinical practice. *Diabetic Medicine*.

[14] Burstal R. J., Reilly J. R., Burstal B. (2018). Fasting or starving? Measurement of blood ketone levels in 100 fasted elective and emergency adult surgical patients at an Australian tertiary hospital. *Anaesthesia and Intensive Care*.

[15] Mahendran Y., Vangipurapu J., Cederberg H. (2013). Association of ketone body levels with hyperglycemia and type 2 diabetes in 9,398 Finnish men. *Diabetes*.

[16] Haidl F., Brabrand M., Henriksen D. P., Lassen A. T. (2015). Lactate is associated with increased 10-day mortality in acute medical patients. *European Journal of Emergency Medicine*.

[17] Kanikaria-Maria P., Jain S. K. (2016). Hyperketonemia and ketosis increase the risk of complications in type 1 diabetes. *Free Radical Biology and Medicine*.

[18] Jain S. K., McVie R. (1999). Hyperketonemia can increase lipid peroxidation and lower glutathione levels in human erythrocytes in vitro and in type 1 diabetic patients. *Diabetes*.

[19] Jain S. K., Kannan K., Lim G., McVie R., Bocchini J. A. (2002). Hyperketonemia increases tumor necrosis factor-*α* secretion in cultured U937 monocytes and type 1 diabetic patients and is apparently mediated by oxidative stress and cAMP deficiency. *Diabetes*.

[20] Rains J. L., Jain S. K. (2011). Hyperketonemia increases monocyte adhesion to endothelial cells and is mediated by LFA-1 expression in monocytes and ICAM-1 expression in endothelial cells. *American Journal of Physiology-Endocrinology and Metabolism*.

[21] Kanikarla-Marie P., Jain S. K. (2015). Hyperketonemia (acetoacetate) upregulates NADPH oxidase 4 and elevates oxidative stress, ICAM-1, and monocyte adhesivity in endothelial cells. *Cellular Physiology and Biochemistry*.

[22] Dennhardt N., Beck C., Huber D. (2016). Optimized preoperative fasting times decrease ketone body concentration and stabilize mean arterial blood pressure during induction of anesthesia in children younger than 36 months: a prospective observational cohort study. *Pediatric Anesthesia*.

[23] Ferrannini E., Mark M., Mayoux E. (2016). CV protection in the EMPA-REG OUTCOME Trial: a “Thrifty Substrate” hypothesis. *Diabetes Care*.

[24] McGuire L. C., Cruickshank A. M., Munro P. T. (2006). Alcoholic ketoacidosis. *Emergency Medicine Journal*.

